# Impact of Acute Exacerbation and Its Phenotypes on the Clinical Outcomes of Chronic Obstructive Pulmonary Disease in Hospitalized Patients: A Cross-Sectional Study

**DOI:** 10.3390/toxics10110667

**Published:** 2022-11-06

**Authors:** Mohammed Kaleem Ullah, Ashwaghosha Parthasarathi, Jayaraj Biligere Siddaiah, Prashant Vishwanath, Swapna Upadhyay, Koustav Ganguly, Padukudru Anand Mahesh

**Affiliations:** 1 Centre for Excellence in Molecular Biology and Regenerative Medicine, Department of Biochemistry, JSS Medical College, JSSAHER, Mysore 570015, Karnataka, India; 2 Global Infectious Diseases Fellow, Division of Infectious Disease and Vaccinology, School of Public Health, University of California, Berkeley, CA 94720, USA; 3 Allergy, Asthma, and Chest Centre, Krishnamurthypuram, Mysore 570004, Karnataka, India; 4 RUTGERS Centre for Pharmacoepidemiology and Treatment Science, New Brunswick, NJ 08901-1293, USA; 5 Department of Respiratory Medicine, JSS Medical College, JSSAHER, Mysore 570015, Karnataka, India; 6 Unit of Integrative Toxicology, Institute of Environmental Medicine (IMM), Karolinska Institutet, 17177 Stockholm, Sweden

**Keywords:** COPD, acute exacerbation, phenotype, AECOPD, biomass, tobacco, mortality

## Abstract

Acute exacerbations of COPD (AECOPD) are clinically significant events having therapeutic and prognostic consequences. However, there is a lot of variation in its clinical manifestations described by phenotypes. The phenotypes of AECOPD were categorized in this study based on pathology and exposure. In our cross-sectional study, conducted between 1 January 2016 to 31 December 2020, the patients were categorized into six groups based on pathology: non-bacterial and non-eosinophilic; bacterial; eosinophilic; bacterial infection with eosinophilia; pneumonia; and bronchiectasis. Further, four groups were classified based on exposure to tobacco smoke (TS), biomass smoke (BMS), both, or no exposure. Cox proportional-hazards regression analyses were performed to assess hazard ratios, and Kaplan–Meier analysis was performed to assess survival, which was then compared using the log-rank test. The odds ratio (OR) and independent predictors of ward admission type and length of hospital stay were assessed using binomial logistic regression analyses. Of the 2236 subjects, 2194 were selected. The median age of the cohort was 67.0 (60.0 to 74.0) and 75.2% were males. Mortality rates were higher in females than in males (6.2% vs. 2.3%). AECOPD-B (bacterial infection) subjects [HR 95% CI 6.42 (3.06–13.46)], followed by AECOPD-P (pneumonia) subjects [HR (95% CI: 4.33 (2.01–9.30)], were at higher mortality risk and had a more extended hospital stay (6.0 (4.0 to 9.5) days; 6.0 (4.0 to 10.0). Subjects with TS and BMS-AECOPD [HR 95% CI 7.24 (1.53–34.29)], followed by BMS-AECOPD [HR 95% CI 5.28 (2.46–11.35)], had higher mortality risk. Different phenotypes have different impacts on AECOPD clinical outcomes. A better understanding of AECOPD phenotypes could contribute to developing an algorithm for the precise management of different phenotypes.

## 1. Introduction

Chronic obstructive pulmonary disease (COPD) is a preventable and treatable condition characterized by non-reversible airflow restriction responsible for significant morbidity, mortality, and healthcare expenditure globally [1,2]. In 2017, there were 545 million chronic respiratory disorders, of which COPD accounted for roughly 50%, with a global prevalence between 9% to 13% [3,4,5]. Sixty-five million individuals worldwide have moderate-to-severe COPD, and it is the third most common cause of death [6]. More than 3.23 million individuals died from COPD in 2019 [7], with low- and middle-income countries (LMICs) suffering 80% of these fatalities [8,9]. Latin America, Sub-Saharan Africa, India, China, and South-East Asia have the highest COPD mortality rates [10]. COPD is the second major cause of non-communicable disease (NCD)-related fatalities in India, which accounts for 18% of the overall global population with COPD [11,12].

Acute exacerbations of COPD (AECOPD) are characterized by an acute aggravation of the underlying chronic inflammation of the airways. They are associated with disease progression, bacterial or viral infections, and exposure to environmental irritants [13]. AECOPD is distinguished by significant airflow obstruction that causes increased labor of breathing and altered gas exchange [14]. Even one exacerbation can result in deteriorating lung function, eventually linked to poor prognosis, prolonged recovery, and lower quality of life [15,16,17]. Lowering the likelihood of future exacerbations is one of the critical objectives of COPD therapy, and clinicians accomplish this by using efficient diagnosis and management strategies. However, the treatment of AECOPD includes various constraints and unknowns that vary depending on each patient’s pathobiological heterogeneity and clinical presentation. Significant heterogeneity in the clinical presentation, risk factors, exposures, and clinical outcomes are present among patients with AECOPD [18,19]. Phenotype refers to the observable combination of disease attributes that describe differences among patients. Several phenotypes of AECOPD have been described previously. These phenotypes include AECOPD-Eosinophilia [20,21,22,23,24,25,26], AECOPD-Bacterial [27,28,29], AECOPD-Pneumonia [30,31,32,33,34,35,36,37,38], and AECOPD-Bronchiectasis [39,40,41]. The results of the MULTI-PHACET study indicate that most patients might have more than one etiology contributing to AECOPD [42]. The etiology, inflammatory biomarkers, clinical presentation, comorbidity, frequency of exacerbations, and other factors have been used to categorize the phenotypes of AECOPD [43].

The two primary environmental risk factors for chronic obstructive pulmonary disease (COPD) worldwide are cigarette smoking and biomass smoke [44]. Tobacco cigarettes are highly processed with numerous inorganic toxic compounds, and biomass smoke is generated from unprocessed organic matter [45]. A systematic review observed that COPD deaths are associated with indoor pollution, and women are affected more than men due to higher rates of exposure to indoor pollution [46]. Biomass smoke exposure in women leads to the development of COPD, as seen in tobacco smokers, with decreased life expectancy and increased mortality [47] but the two causes differ in the pathophysiological changes made to the lungs due to the difference in chemical compositions between tobacco and biomass. Females with biomass-exposed COPD are associated with more air-trapping, anthracosis, and pulmonary fibrosis, and less emphysema, compared to tobacco-smoking COPD [48].

Most of these studies were performed in developed countries such as those in Europe and North America [49]. There is a need to understand the various AECOPD phenotypes in LMIC countries and their associated clinical outcomes such as mortality, need for ICU admission, and length of hospital stay. It is equally essential to understand gender-based differences in AECOPD outcomes and the effect of different types of exposure, such as tobacco smoking and biomass fuel exposure, on AECOPD phenotypes and outcomes. This study aimed to assess differences in clinically significant outcomes such as mortality, need for ICU admission, and length of hospital stay between various AECOPD phenotypes grouped according to the pathology or risk factors such as tobacco smoking and biomass smoke exposure.

## 2. Materials and Methods

### 2.1. Cohort Description

This cross-sectional study was conducted in a tertiary care university teaching hospital—JSS Medical College and Hospital, Mysore, India—by reviewing the subjects’ hospital records. The eligible subjects among the patients admitted between 1 January 2016 to 31 December 2020 were identified by the ICD-10 (International Classification of Diseases, tenth edition) codes J44.1 and J44.9. A total of 2194 AECOPD subjects were included in the study, constituting the Mysuru COPD (MYCO) Cohort. During the study period, age, gender, comorbidities, length of hospital stay, admission to ward or ICU, outcome (survived/expired), and hematological investigations (neutrophils, lymphocytes, eosinophils, monocytes, basophils, RBC count, and platelet count) were documented and recorded from the hospital records. NLR (Absolute neutrophil count/Absolute lymphocyte count) and Charlson’s comorbidity score were calculated. Any additional secondary diagnoses were defined according to the ICD-10 coding system. Subjects’ demographic and clinical characteristics are presented in Table 1 based on pathology and Table 2 based on exposure.

### 2.2. Definitions

COPD is a common, preventable, treatable disease characterized by persistent respiratory symptoms and airflow limitation due to airway and/or alveolar abnormalities, usually caused by significant exposure to noxious particles or gases and influenced by host factors including abnormal lung development [2]. AECOPD is defined as acute worsening of respiratory symptoms (such as increase in coughing, quantity of sputum, breathlessness, or increase in purulence of sputum) that results in additional therapy [50]. Cor pulmonale, pulmonary hypertension, and other additional diagnoses are defined according to the ICD10 coding system. Charlson’s comorbidity index was calculated as a composite risk of all associated comorbidities, where a higher score predicts a higher risk of mortality or higher resource-use [51].

Bacterial infection was defined as the presence of positive sputum culture for pathogenic bacteria. Eosinophilia was defined as the presence of 3% or more eosinophils in the differential leukocyte count [52]. The presence of an ICD-10 code identified pneumonia and bronchiectasis subjects.

Smokers are defined as adult smokers with a smoking history of >10 pack years. The burning of various fuels, including wood, animal dung, and crop wastes, to produce the energy required for heating and cooking in many houses worldwide results in the production of biomass smoke [53]. Biomass exposure is defined as chronic exposure to biomass smoke for at least ten years [54,55]. Non-tobacco smoke and non-biomass smoke COPD might be due to causes other than smoking and biomass exposure, such as prolonged exposure to dust due to dusty occupations such as agricultural farming, sericulture farming, working in the cotton industry, chronic asthma, and post-TB sequelae.

The common reasons for hospitalization of AECOPD subjects in the general ward were failed response to initial medical management, severe symptoms, serious comorbidities, the onset of new physical signs, and acute respiratory failure (without using accessory respiratory muscles). Common symptoms for admission to respiratory or medical ICU are very severe symptoms, acute respiratory failure (with accessory respiratory muscle), persistent or worsening hypoxemia and/or severe respiratory acidosis, need for non-invasive or invasive mechanical ventilation, and hemodynamic instability [50].

The subjects were categorized based on pathology and exposure as below. Pathology phenotypes (six groups):

1. AECOPD-Non-bacterial and non-eosinophilic (NBE) group, consisting of AECOPD subjects who had no bacterial infection and no peripheral blood eosinophilia;
2. AECOPD-Bacterial (B) group, consisting of subjects who had bacterial AECOPD without eosinophilia;
3. AECOPD-Eosinophilia (E) group, consisting of AECOPD subjects who had peripheral blood eosinophilia and no bacterial infections;
4. AECOPD-Bacterial with eosinophilia (BE) group, consisting of subjects who had bacterial AECOPD with peripheral blood eosinophilia;
5. AECOPD-Pneumonia (P) group, consisting of AECOPD subjects with pneumonia;
6. AECOPD-Bronchiectasis (BC) group, consisting of AECOPD subjects with bronchiectasis.

Exposure groups (four groups):

1. Non-tobacco smoke and non-biomass smoke (NTS and NBMS) AECOPD group, consisting of AECOPD subjects who were non-smokers with no biomass exposure;
2. Tobacco smoke (TS) AECOPD group, consisting of AECOPD subjects who were smokers;
3. Biomass smoke (BS) AECOPD group, consisting of AECOPD subjects who were exposed to biomass smoke;
4. Tobacco smoke and biomass smoke (TS and BMS) AECOPD group, consisting of AECOPD subjects who were smokers with biomass exposure.

The subjects eligible were identified by the ICD-10 codes J44.1 and J44.9. Male and female subjects ≥ 40 years of age diagnosed with acute exacerbation of COPD were included in the study. Subjects with relevant missing data were excluded from the study.

### 2.3. Statistical Analysis

Statistical analysis was performed employing Jamovi (v1.6, The Jamovi project, SYD, AUS). Data were checked for normality of distribution using Shapiro–Wilk’s test. If they were normally distributed, continuous variables were presented as mean ± standard deviation. If not, they were presented as median with their interquartile range. Categorical variables were presented as percentages. Statistical significance was assessed by the Chi-square test for categorical variables and by the Kruskal–Wallis test for continuous variables and expressed with medians and interquartile ranges (IQRs). Cox proportional-hazards regression analyses were performed to assess univariate and multivariate hazard ratios. The Kaplan–Meier method was used to draw survival curves, while the survival rates were compared using the log-rank test. The odds ratio (OR) and independent predictors of type of ward admission and length of hospital stay were assessed by binomial logistic regression analyses. A two-tailed *p*-value of <0.05 was considered statistically significant.

## 3. Results

**Figure 1:**
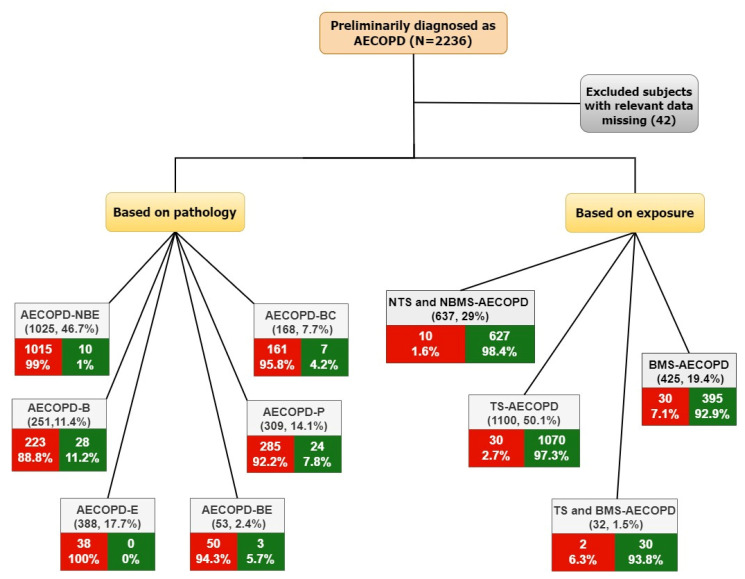
Flowchart of the study describing the categorization of AECOPD phenotypes based on exposure and pathology and their mortality. Red: Expired, Green: Alive, AECOPD: Acute exacerbation of chronic obstructive pulmonary diseases, NBE: No bacterial infection and no eosinophilia, B: Bacterial infection, E: Eosinophilia and no bacterial infections, BE: Bacterial infection with eosinophilia, P: Pneumonia, BC: Bronchiectasis, NTS and NBMS: Non-tobacco smoke and non-biomass smoke AECOPD, TS: Tobacco smoke, BMS: Biomass smoke, TS and BMS: Tobacco smoke and biomass smoke.

During the five-year study period, 2194 AECOPD patients met the inclusion criteria and were categorized into six groups based on pathology; the most common group was the AECOPD-NBE group (n = 1025, 46.7%), followed by the AECOPD-E group (n = 388, 17.7%), AECOPD-P group (n = 309, 14.1%), AECOPD-B group (n = 251, 11.4%), AECOPD-BC group (n = 168, 7.7%), and AECOPD-BE group (n = 53, 2.4%). Patients were further categorized into four groups based on exposure; the most common group was TS-AECOPD (n = 1100, 50.1%), followed by NTS and NBMS-AECOPD (n = 637, 29%), BMS-AECOPD (n = 425, 19.4%), and TS and BMS-AECOPD (n = 32, 1.5%) (Figure 1). The median (IQR) age of the subjects was 67.0 (60.0 to 74.0). Males accounted for 75.2% of the study subjects. Among the common comorbidities documented, diabetes mellitus (23.8%) was the most common, followed by heart disease (14.9%) and renal disease (8.5%), and when stratified based on sex, females had higher rates of diabetes mellitus (30.6; *p* < 0.001) while males had higher incidence of heart diseases (15.6%; *p* = 0.09) and renal diseases (3.2%; *p* = 0.007) (Table S1).

The AECOPD-BC group consisted of younger subjects [65 (56 to 71)] compared to the other groups (*p* < 0.016). The AECOPD-BC group had a higher proportion of females (36.9%) compared to the other groups (*p* < 0.001). A longer duration of hospital stay (*p* < 0.001) was observed in AECOPD subjects with bacterial infection (AECOPD-B group and AECOPD-BE group) and pneumonia (AECOPD-P group). The highest incidence of ICU admissions were observed in the AECOPD-B group (51%), and the lowest were observed in the AECOPD-E group (2.1%) (*p* < 0.001). Higher mortality was observed in the AECOPD-B group (11.2%), and no mortality was observed in the AECOPD-E group (0%) (*p* < 0.001). A total of 54.6% of cases observed with AECOPD-NBE were smokers, followed by AECOPD-P (53.4%); AECOPD-BC had higher incidence of BMS-AECOPD (30.4%), followed by AECOPD-BE (24.5%). In the case of TS and BMS-AECOPD, it was highest in AECOPD-BC (6%) followed by AECOPD-BE (3.8%) (*p* < 0.001) (Table 1).

Overall, mortality was higher in females when compared to males (6.2% vs. 2.3%, respectively; *p* < 0.001) (Table S1), and in all the different phenotypes, higher mortality in females was observed than in males. This was such for cases of AECOPD-NBE (2.7% vs. 0.5%, respectively; *p* = 0.003), AECOPD-P (11.4% vs. 6.5%, respectively; *p* = 0.163), AECOPD-BC (6.5% vs. 2.8%, respectively; *p* = 0.257), AECOPD-B (21% vs. 7.9%, respectively; *p* = 0.005), and AECOPD-BE (15.4% vs. 2.5%, respectively; *p* = 0.081) (Table S2). A total of 27.5% of AECOPD-P patients had bacterial pneumonia, and the remaining cases were non-bacterial pneumonia. Of the AECOPD-BC group, 25.6% of patients had bacterial infections and the remaining patients had non-bacterial bronchiectasis. A higher NLR ratio (*p* < 0.001) was observed in the AECOPD-P and AECOPD-B groups, and the lowest NLR ratio was observed in the AECOPD-E group. The AECOPD-E group had significantly higher lymphocyte [20.4 (14.2 to 27.5); *p* < 0.001], eosinophil [4.90 (3.9 to 6.73); *p* < 0.001], monocyte [5.0 (3.9 to 6.2); *p* < 0.001], and basophil [0.4 (0.3 to 0.7); *p* < 0.001] counts than any other phenotypes. The AECOPD-BE group had a significantly higher RBC count [4.70 (4.35 to 5.05); *p* = 0.002]. The AECOPD-B [8.50 (4.6 to 17.6); *p* < 0.001] and AECOPD-P [8.50 (4.7 to 17.8); *p* < 0.001] groups had significantly higher neutrophil–lymphocyte ratios. The AECOPD-P group had significantly lower lymphocyte [9.6 (5.05 to 16.4); *p* < 0.001], monocyte [3.95 (2.4 to 5.45); *p* < 0.001], and RBC [4.46 (4.0 to 4.97); *p* = 0.002] counts. The AECOPD-B group had significantly lower eosinophils [0.2 (0.0 to 1.2); *p* < 0.001]. The AECOPD-E group had a significantly lower neutrophil–lymphocyte ratio [3.3 (2.2 to 5.30); *p* < 0.001] (Table 1).

The TS and BMS-AECOPD group consisted of younger subjects [65 (61.5 to 68.3)] compared to other groups (*p* < 0.01). The BMS-AECOPD group had a higher proportion of females (93.4%) compared to other groups (*p* < 0.01). A longer duration of hospital stay (*p* = 0.019) was observed in the BMS-AECOPD group. The TS-AECOPD group had a significantly higher neutrophil–lymphocyte ratio [6.10 (3.5 to 13.2); *p* < 0.002], while BMS-AECOPD had higher lymphocyte [15.25 (9.6 to 22.1); *p* < 0.001] and platelet count [2.65 (2.16 to 3.32); *p* < 0.001]; TS and BMS-AECOPD had higher eosinophil [2.4 (0.4 to 3.9); *p* < 0.001] and RBC count [4.83 (4.6 to 5.3); *p* < 0.001], and a higher neutrophil–lymphocyte ratio [6.10 (3.0 to 13.4); *p* < 0.002]. The highest mortality was observed in the BMS-AECOPD group (7.1%) and the lowest mortality in the NTS and NBMS-AECOPD group (1.6) (*p* < 0.01) (Table 2). When the type of exposure was stratified based on gender, we observed a significant difference between male and female mortality in NTS and NBMS-AECOPD (1.0% vs. 3.6%, respectively; *p* = 0.030) and BMS-AECOPD (3.6% vs. 7.3%, respectively; *p* = 0.456). Still, in TS-AECOPD, higher mortality was observed in males (2.7% vs. 0%, respectively; *p* = 0.614) and the TS and BMS-AECOPD group had only male patients, with a 6.3% mortality rate (Table S3).

**Figure 2:**
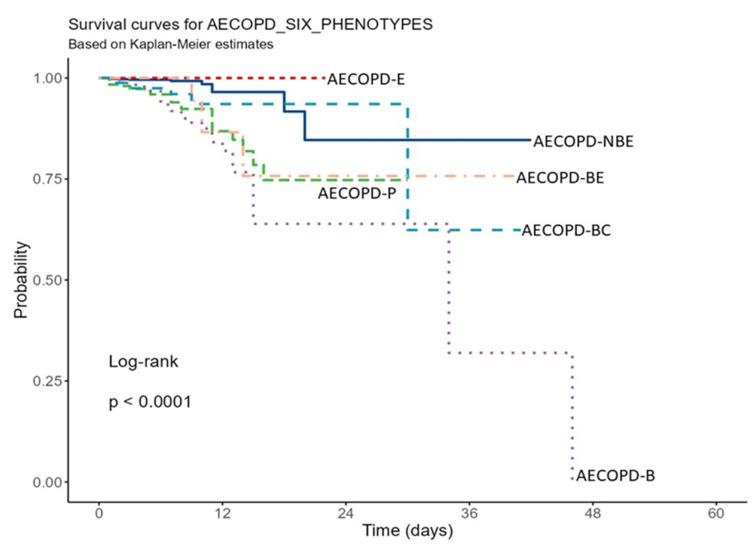
Kaplan–Meier survival curve for phenotypes based on pathology. AECOPD: Acute exacerbation of chronic obstructive pulmonary diseases, NBE: No bacterial infection and no eosinophilia, B: Bacterial infection, E: Eosinophilia and no bacterial infections, BE: bacterial infection with eosinophilia, P: Pneumonia, and BC: Bronchiectasis.

A significant difference between the six groups was observed following Kaplan–Meier analysis using the log-rank test (*p* < 0.0001) (Figure 2), which showed that the AECOPD-B group had a significantly higher risk of mortality compared to the other groups. A pairwise comparison was also performed for each group (Table 3). We observed that intergroup comparisons for survival among conditions associated with bacterial infections such as AECOPD-B, AECOPD-BE, AECOPD-P, and AECOPD-BC were not statistically significant. Intergroup comparisons for survival between conditions related to bacterial infections (AECOPD-B, AECOPD-BE, AECOPD-P, and AECOPD-BC) and those without bacterial infections (AECOPD-E and AECOPD-NBE) were statistically significant, except for AECOPD-BE with AECOPD-NBE.

**Figure 3:**
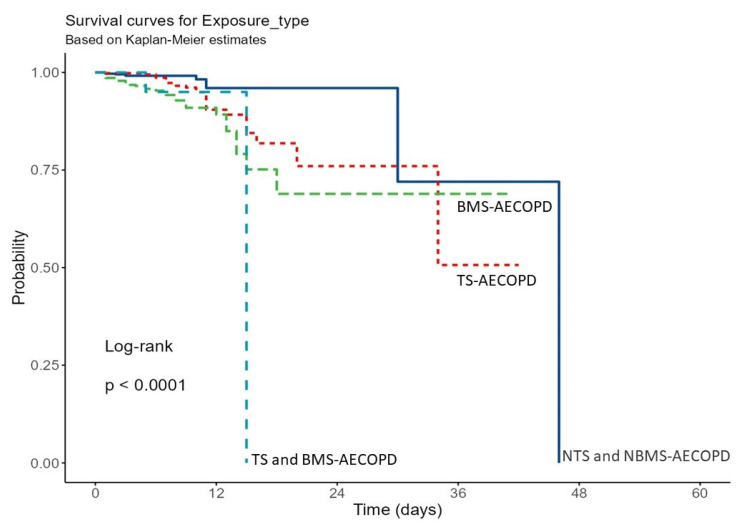
Kaplan–Meier survival curve for phenotypes based on exposure. Definition of abbreviations: AECOPD: Acute exacerbation of chronic obstructive pulmonary diseases, NTS and NBMS: Non-tobacco smoke and non-biomass smoke, TS: Tobacco smoke, BMS: Biomass smoke, and TS and BMS: Tobacco smoke and biomass smoke.

A significant difference between the four groups was observed following Kaplan–Meier analysis using the log-rank test (*p* < 0.0001), which showed that the BMS-AECOPD group had a significantly higher risk of mortality compared to the other groups (Figure 3). A pairwise comparison was also performed for each group (Table 4). Three intergroup comparisons for survival were significant: BMS-AECOPD vs. NTS and NBMS-AECOPD, BMS-AECOPD vs. TS-AECOPD, and TS and BMS-AECOPD vs. NTS and NBMS-AECOPD.

Two models were considered to evaluate factors with independent association to mortality. Model 1 assessed exposures and model 2 considered only phenotypes based on pathology (Table 5). Univariate Cox proportional-hazard regression analysis for survival in model 1 showed that females, those older than 60, those with type 2 respiratory failure, patients with sepsis, and the AECOPD-B, AECOPD-P, and AECOPD-BC groups were associated with poor survival. On multivariate Cox proportional-hazard regression analysis, females [Hazards ratio (HR) (95% Confidence Interval (CI): 3.05 (1.83–5.09)], type 2 respiratory failure [HR (95% CI: 1.74 (1.04–2.91)], sepsis [HR (95% CI: 3.13 (1.72–5.71)], AECOPD-B group membership [HR 95% CI 6.42 (3.06–13.46,)], AECOPD-BE group membership [HR (95% CI: 3.84 (1.04–14.20)], AECOPD-P group membership [HR (95% CI: 4.33 (2.01–9.30)], and AECOPD-BC group membership [HR (95% CI: 2.72 (1.00–7.38)] were found to be independently associated with poor survival (Model 1: Table 5).

Univariate Cox proportional-hazard regression analysis for survival in model 2 showed that age higher than 60, type 2 respiratory failure, patients with sepsis, TS-AECOPD, BMS-AECOPD, and TS and BMS-AECOPD were associated with poor survival. On multivariate Cox proportional-hazard regression analysis, type 2 respiratory failure [HR (95% CI: 1.75 (1.04–2.93)], sepsis [HR (95% CI: 5.23 (2.96–9.25)], TS-AECOPD [HR 95% CI 2.17 (1.02–4.63)], BMS-AECOPD [HR 95% CI 5.28 (2.46–11.35)], and TS and BMS-AECOPD [HR 95% CI 7.24 (1.53–34.29)] were found to be independently associated with poor survival (Model 2: Table 5). Since the exposure was already segregated by gender—99.2% of TS-AECOPD subjects were males and 93.4% of BMS-AECOPD subjects were females—gender was not used in model 2.

On binomial logistic regression analyses, AECOPD-B [10.66 (95% CI: 7.65–14.87)], AECOPD-P [5.56 (95% CI: 4.04–7.6)], AECOPD-BE [4.32 (95% CI: 2.30–8.13)], AECOPD-BC [3.341 (95% CI: 2.18–5.11)], and CCI [1.27 (95% CI: 1.14–1.42)] were found to be strong independent predictors of type of ward admission (Table S4). On binomial logistic regression analyses, AECOPD-BE [2.29 (95% CI: 1.29–4.06)], AECOPD-B [2.271 (95% CI: 1.69–3.05)], AECOPD-P [2.09 (95% CI: 1.58–2.75)], AECOPD-BC [1.45 (95% CI: 0.99–2.09)], gender [1.44 (95% CI: 1.16–1.78)], and CCI [1.20 (95% CI: 1.10–1.31)] were found to be strong independent predictors of length of hospital stay (Table S5).

## 4. Discussion

We observed six phenotypes of acute exacerbation of COPD which impact clinical outcomes. To the best of our knowledge, this is the first attempt to compare the various AECOPD phenotypes based on pathology and exposure, their characteristics, and their clinical outcome in an LMIC country. We observed an overall mortality rate of 3.3%. We observed the highest mortality in AECOPD-B (HR: 6.42), followed by AECOPD-P (HR:4.33). The highest ICU admissions were for AECOPD-B (51%) and AECOPD-P (35.6%). Besides AECOPD-E and AECOPD-BC, which had lower Charlson’s comorbidity indices, comorbidities were similar across various phenotypes. Among subjects with AECOPD, combined TS and BMS-AECOPD (HR 7.24) was observed to have a greater mortality risk than BMS-AECOPD (HR 5.28), followed by TS-AECOPD (HR 2.17), and an additive effect was observed. Female patients with AECOPD had a greater risk of death and poor outcome than male patients.

Bacterial infection associated with COPD exacerbation, pneumonia associated with COPD exacerbation, and sepsis are the risk factors most closely associated with a greater risk of death. Lower respiratory tract infections, both acute and chronic, are frequent in patients with AECOPD. Infections contribute considerably to these patients’ poor clinical course and overall morbidity and mortality [56,57]. AECOPD patients have an impaired response to microbial colonization and infection. These impaired responses, such as impaired alveolar macrophage and toll-like receptor activity, increase infection vulnerability and promote an oxidative stress response that injures lung tissue [58,59]. Bacterial infections are responsible for more than 50% of AECOPD exacerbations [60]. Bacterial load in the lungs of AECOPD patients is an essential determinant of airway inflammation. Increased concentration of pathogens is directly correlated with greater intensity of neutrophilic airway inflammation [60,61]. Another model of exacerbation pathogenesis suggests that acquiring novel bacterial strains is a major driver of acute exacerbations [56,62]. Among different AECOPD phenotypes, the highest mortality rate was observed in the AECOPD-B phenotype (11.2%), with the highest hazard ratio of 6.42 compared to other AECOPD phenotypes. Other studies evaluating mortality rates in AECOPD with bacterial infections range from 2.4% to 8.2% [27,28]. The AECOPD-B phenotype was also associated with a longer duration of hospitalization, and had the highest risk of ICU admissions compared to other AECOPD phenotypes. Other studies evaluating the length of hospital stay ranged in their evaluation from 10 to 13 days [27,28,29], but had fewer ICU admissions than our study, with 51% of AECOPD-B patients admitted to ICU.

Pneumonia is a significant cause of hospitalization and mortality, especially among AECOPD patients, and is linked to abnormal host-defense mechanisms [63,64,65]. A study by Gutierrez et al. analyzed sputum samples from pneumonia patients, and compared them with AECOPD patients and patients with both pneumonia and AECOPD. They observed that the microenvironment present in the lung modulates the activation of macrophages, which may lead to disparities in cytokine generation and specific macrophage activation when AECOPD and pneumonia are present simultaneously; this can cause different inflammatory responses leading to different outcomes [65]. Furthermore, a study by Huerta et al. found that, compared to other cohorts, patients with AECOPD-P had a higher level of Interleukin 6, tumor necrosis factor-α, C-reactive protein, and procalcitonin [30]. Our cohort’s prevalence of AECOPD-P was 14.1%, and they had the second highest mortality rate at 7.8%. Various studies have shown that AECOPD-P has mortality rates ranging from 3.4% to 13.2% [30,31,32,33,34,35,36]. In our study, AECOPD subjects with pneumonia had a significantly higher mortality risk than AECOPD-NBE and AECOPD-E as assessed by Kaplan–Meier analysis (30-day mortality). Similar results were also seen in other studies investigating AECOPD-P [37,38]. AECOPD-P had a longer duration of hospitalization, between 4 to 10 days, which was twice that of AECOPD-NBE. The length of hospitalization in other studies varied from 7 days to 16 days [30,31,32,33,34]. The percentage of ICU admission was significantly lower in previous studies, ranging from 2.6% to 26%, compared to our study (35.6%), which could be due to a higher percentage of sepsis patients (17.2%) among the AECOPD-P phenotype in our study. According to several studies, sepsis is more likely to occur in AECOPD patients due to corticosteroids, underlying comorbidities, and possibly compromised barrier function, leading to an increased risk of developing pneumonia [66,67,68]. A study performed by Chen et al. studied the impact of sepsis on COPD patients’ outcomes, and they identified that COPD patients with sepsis had a higher risk of pneumonia, severe exacerbations, and mortality compared to COPD patients without sepsis [69].

The role of eosinophilic inflammation in the exacerbation of COPD is not clear. Subjects with AECOPD and eosinophilia have better corticosteroid responsiveness [70,71,72] and eosinophilic AECOPD patients have better outcomes [23]. The prevalence of AECOPD-E in our cohort was 17.7%, with zero mortality, and these patients had the best outcomes compared to other phenotypes. Several studies have observed lower mortality rates in eosinophilic patients ranging from 0% to 1% [20,21,22,23] similar to our study, which could be due to fewer comorbidities [73], shorter length of hospital stay (2 days to 8.81 days) [24,25,74,75], and less-frequent admissions [75,76,77] in eosinophilic patients than other phenotypes. The number of ICU admissions ranged from 1.32% to 11.7% [21,24,26,78,79,80], similar to our study, which had ICU admission in 2.1% of subjects with AECOPD-E.

Subjects with AECOPD and both eosinophilia and bacterial infection (AECOPD-BE) formed the smallest group (2.4%) in our study, with a mortality rate of 5.7%. It had the third highest ICU admission (30.2%) with odds of 4.33 (95% CI: 2.3–8.13) and the length of hospital stay ranged from 4 days to 9 days with odds of 2.29 (95% CI: 1.29–4.06) compared to AECOPD-NBE. We could not find any studies showing a direct relationship between AECOPD and bacterial infection and eosinophilia. Conversely, we found several studies showing an inverse relationship between eosinophil count and bacterial load, suggesting that the presence of less than 2% eosinophils in AECOPD events may indicate bacterial infection [81,82,83]. It is unclear what causes the drop in eosinophil levels during bacterial infection. Still, possible reasons might be the adrenal glucocorticoid activation in reaction to the stress of bacterial infection, or the rapid accumulation of eosinophils at the inflammatory site possibly causing a decrease in the number of eosinophils in circulation [84,85]

Mitochondrial DNA which plays a vital role in regulating cellular metabolism, signaling pathways, and cell death [86] can be released by eosinophils as an innate immune response to pathogens to form extracellular mitochondrial DNA traps and express specific pattern-recognition receptors, such as Toll-like receptor 4, which can recognize pathogens and trigger an immune response [87,88]. Eosinophil granule proteins also possess bactericidal activity [89]. Furthermore, high levels of cell-free mitochondrial DNA have been found in the plasma of former smokers affected by COPD [90], in the serum of mice that developed emphysema induced by chronic exposure to cigarette smoke for 6 months [90], and also in the bronchoalveolar lavage of mice acutely exposed to cigarette smoke [91]. In addition, in a small single-center study, high levels of total cell-free DNA were detected in the plasma of patients with COPD exacerbations admitted to the hospital, and were associated with an increased risk of 5-year mortality [92]. It has been also shown that cells other than eosinophils could release mitochondrial DNA, including neutrophils [93]. In the context of COPD and its main risk factor (cigarette smoke), it has been found that human bronchial alveolar cells exposed to cigarette smoke release mitochondrial DNA by extracellular vesicles and cells debris, and this, in turn, may trigger the upregulation of typical proinflammatory cytokines observed in COPD [90].

Bronchiectasis is highly prevalent in AECOPD patients, ranging from 4% to 72% [94]. It is associated with severe bronchial inflammation, higher functional impairment, higher frequency and severity of exacerbations, and increased hospitalization in AECOPD patients [95,96,97]. The prevalence of AECOPD-BC in our study was 7.7%, with a mortality of 4.2%. We observed a similar length of hospital stay (4 to 8 days) in our AECOPD-BC cohort compared to other studies, which ranged from 5.7 days to 9.6 days [39,40,41]. Sánchez-Muñoz et al. observed 5.5% ICU admissions in the AECOPD-BC cohort [41] in contrast, in our study, we observed 23.2% ICU admissions in the AECOPD-BC cohort. Our study found that the AECOPD-BC phenotype was higher in female than in male patients, similar to observations made in other similar studies [98,99,100,101].

One of the important risk factors for non-smoking COPD is exposure to biomass fuels [102], which are critical for activities of daily living such as cooking and heating, especially in developing nations [103]; almost 3 billion people worldwide use biomass fuels as their primary source of energy. Biomass fuels account for 50–90% of household energy in many developing countries. Biomass combustion fumes contain several pollutants with potential to cause long-term damage to the lungs. This includes particulate matter, volatile organic compounds such as and formaldehyde and benzene, and other organic matter, including polycyclic aromatic hydrocarbons such as benzopyrene [54]. The US Environmental Protection Agency has established National Ambient Air Quality Standards, and the recommended daily average of PM_10_ is below 150 μg m^−3^. The recommended annual average is below 50 μg m^−3^. In homes that use biomass fuels, the average PM_10_ concentrations vary between 200 to 5000 μg m^−3^, depending on the presence of a chimney, ventilation (windows, cooking with doors open), and the type of fuel and stove. Cigarette smoke contains more than 4000 chemicals, including many toxic compounds and well-known risk factors for developing COPD [44,104,105,106,107].

We observed the highest mortality rates in BMS-AECOPD (7.1%), followed by TS and BMS-AECOPD (6.3%). Various studies have shown mortality rates for TS-AECOPD ranging from 6.8% to 29% [47,108,109,110,111,112,113]; for BMS-AECOPD, ranging from 8.4% to 18% [47,112]; and for NTS and NBMS-AECOPD, ranging from 3.8% to 24.67% [108,109,110,113]. Biomass exposure was predominantly seen in females, and included 93.4% of females and 6.6% of males. Among females exposed to biomass, mortality rates were 7.3% versus 3.6% among males exposed to biomass. A higher number of subjects with BMS-AECOPD had diabetes mellitus, hypertension, obesity, obstructive sleep apnea, and renal failure than subjects with TS and BMS-AECOPD, who were all males. Female gender was an independent risk factor for AECOPD-related mortality in our study, and few reasons have been identified from previous studies. Females have smaller airways and lungs [114]; there are gender differences in lung physiology and microbiota composition, which may influence the severity and progression of chronic respiratory disease states [100,115]; the respiratory microbiome is vulnerable to various host immunological and inflammatory effects, and seems to have sex-specific characteristics [116,117]; and chronic persistent inflammation is thought to be more harmful in females, increasing tissue damage and worsening illness severity [118]. The gender divide in human health and disease could be primarily explained by variations in genetics and sex steroid hormones, both in kind and concentration [119].

There is a steady decline in lung function with advancing age, which increases the risk of dyspnea and the prevalence of chronic pulmonary disorders in older people [120,121]. The prevalence of AECOPD is two to three times higher in patients older than 60. With increasing age, comorbidities also increase, but these comorbidities manifest earlier in AECOPD patients than in non-COPD patients [122]. The aging process in the lungs and AECOPD shares many similarities. Many signs of aging are present in AECOPD, indicating that accelerated aging may be a pathogenic factor in AECOPD [123].

To our knowledge, this is one of the most extensive studies in India on the survival of different phenotypes of AECOPD based on both pathologies and exposure. The study’s main drawback is that it has a monocentric design, making it harder for our findings to generalize. Only routine bacterial cultures were performed for bacterial identification. Other causes of AECOPD, such as viral AECOPD, were not investigated. Sputum induction was not performed; therefore, further characterization of AECOPD could not be achieved. In the future, more advanced techniques—such as the Biofire test—to identify viruses, bacteria, parasites, yeast, and antimicrobial resistance genes, in addition to sputum induction, can help to identify AECOPD phenotypes with even greater resolution. 

## 5. Conclusions

In conclusion, we observed significant gender differences, with females having a much higher mortality risk than males. AECOPD-B patients, followed by AECOPD-P patients, were at higher mortality risk and had a more-extended hospital stay. Subjects with biomass exposure and who smoked, followed by subjects with biomass exposure, were at higher mortality risk. Different phenotypes have different impacts on AECOPD clinical outcomes. A better understanding of AECOPD phenotypes could contribute to developing an algorithm for the precise management of different phenotypes.

## Figures and Tables

**Table 1:** Clinical characteristics of AECOPD phenotypes categorized based on pathology.

		Total (N = 2194)	AECOPD-NBE (N = 1025)	AECOPD-B (N = 251)	AECOPD-E (N = 388)	AECOPD-BE (N = 53)	AECOPD-P(N = 309)	AECOPD-BC (N = 168)	*p*-Value
Age in years	Median (IQR)	67.0 (60.0 to 74.0)	67.0 (60.0 to 74.0)	66.0 (60.0 to 74.5)	66.0 (59.0 to 74.0)	70.0 (59.0 to 75.0)	67.0 (60 to 75.0)	65.0 (56 to 71.0)	0.016 *
Gender	Male (n, %)	1649 (75.2)	805 (78.5)	189 (75.3)	279 (71.9)	40 (75.5)	230 (74.4)	106 (63.1)	<0.001 ^†^
	Female (n, %)	545 (24.8)	220 (21.5)	62 (24.7)	109 (28.1)	13 (24.5)	79 (25.6)	62 (36.9)	
LOS in days	Median (IQR)	5.0 (3.0 to 8.0)	5.0 (3.0 to 7.0)	6.0 (4.0 to 9.5)	4.0 (3.0 to 6.0)	6.0 (4.0 to 9.0)	6.0 (4.0 to 10.0)	5.0 (4.0 to 8.0)	<0.001 *
Admission to Ward/ICU	Ward (n, %)	1794 (81.8)	926 (90.3)	123 (49.0)	380 (97.9)	37 (69.8)	199 (64.4)	129 (76.8)	<0.001 ^†^
	ICU (n, %)	400 (18.2)	99 (9.7)	128 (51.0)	8 (2.1)	16 (30.2)	110 (35.6)	39 (23.2)	
Hospital outcome	Alive (n, %)	2122 (96.7)	1015 (99)	223 (88.8)	388 (100)	50 (94.3)	285 (92.2)	161 (95.8)	<0.001 ^†^
	Dead (n, %)	72 (3.3)	10 (1.0)	28 (11.2)	0 (0)	3 (5.7)	24 (7.8)	7 (4.2)	
Bacterial infection	Yes (n, %)	432 (19.7)	0 (0)	251 (100)	0 (0)	53 (100)	85 (27.5)	43 (25.6)	<0.001 ^†^
Alcohol consumption	Yes (n, %)	310 (14.1)	170 (16.6)	21 (8.4)	52 (13.4)	2 (3.8)	50 (16.2)	15 (8.9)	0.001 ^†^
NTS and NBMS-AECOPD	Yes (n, %)	637 (29.0)	295 (28.8)	75 (29.9)	128 (33.0)	17 (32.1)	76 (24.6)	46 (27.4)	<0.001 ^†^
TS-AECOPD	Yes (n, %)	1100 (50.1)	560 (54.6)	114 (45.4)	179 (46.1)	21 (39.6)	165 (53.4)	61 (36.3)	
BMS-AECOPD	Yes (n, %)	425 (19.4)	164 (16.0)	54 (21.5)	77 (19.8)	13 (24.5)	66 (21.4)	51 (30.4)	
TS and BMS-AECOPD	Yes (n, %)	32 (1.5)	6 (0.6)	8 (3.2)	4 (1.0)	2 (3.8)	2 (0.6)	10 (6.0)	
CCI	Median (IQR)	4.0 (3.0 to 5.0)	4.0 (3.0 to 5.0)	4.0 (3.0 to 5.0)	3.0 (2.0 to 4.0)	4.0 (3.0 to 5.0)	4.0 (3.0 to 5.0)	3.0 (2.0 to 4.0)	<0.001 *
**Comorbidities**									
Diabetes mellitus	Yes (n, %)	522 (23.8)	249 (24.3)	60 (23.9)	88 (22.7)	14 (26.4)	77 (24.9)	34 (20.2)	0.850 ^†^
Heart diseases	Yes (n, %)	327 (14.9)	162 (15.8)	37 (14.7)	53 (13.7)	9 (17.0)	45 (14.6)	21 (12.5)	0.834 ^†^
Renal diseases	Yes (n, %)	186 (8.5)	86 (8.4)	26 (10.4)	20 (5.2)	5 (9.4)	41 (13.3)	8 (4.8)	0.002 ^†^
Liver diseases	Yes (n, %)	56 (2.6)	29 (2.8)	13 (5.2)	5 (1.3)	1 (1.9)	7 (2.3)	1 (0.6)	0.028 ^†^
Cor pulmonale	Yes (n, %)	452 (20.6)	217 (21.2)	62 (24.7)	57 (14.7)	5 (9.4)	63 (20.4)	48 (28.6)	<0.001 ^†^
Hypertension	Yes (n, %)	758 (34.5)	362 (35.3)	88 (35.1)	140 (36.1)	23 (43.4)	100 (32.4)	45 (26.8)	0.176 ^†^
Obesity	Yes (n, %)	129 (5.9)	61 (6.0)	14 (5.6)	33 (8.5)	7 (13.2)	12 (3.9)	2 (1.2)	0.002 ^†^
OSA	Yes (n, %)	137 (6.2)	64 (6.2)	12 (4.8)	37 (9.5)	7 (13.2)	16 (5.2)	1 (0.6)	<0.001 ^†^
PAH	Yes (n, %)	383 (17.5)	163 (15.9)	49 (19.5)	65 (16.8)	17 (32.1)	46 (14.9)	43 (25.6)	0.001 ^†^
Sepsis	Yes (n, %)	94 (4.3)	19 (1.9)	15 (6.0)	1 (0.3)	0 (0)	53 (17.2)	6 (3.6)	<0.001 ^†^
T1RF	Yes (n, %)	80 (3.6)	35 (3.4)	12 (4.8)	13 (3.4)	1 (1.9)	15 (4.9)	4 (2.4)	0.595 ^†^
T2RF	Yes (n, %)	371 (16.9)	166 (16.2)	61 (24.3)	30 (7.7)	14 (26.4)	69 (22.3)	31 (18.5)	<0.001 ^†^
Lymphocytes %	Median (IQR)	13.5 (7.3 to 21)	12.6 (6.7 to 19.3)	10.0 (5.1 to 16.5)	20.4 (14.2 to 27.5)	18.1 (11.0 to 25.7)	9.6 (5.05 to 16.4)	14.5 (8.15 to 19.8)	<0.001 *
Eosinophils %	Median (IQR)	1.05 (0.2 to 3.3)	0.5 (0.1 to 1.3)	0.2 (0.0 to 1.2)	4.90 (3.9 to 6.73)	4.40 (3.8 to 5.50)	0.5 (0.1 to 1.85)	0.9 (0.2 to 3.0)	<0.001 *
Monocytes %	Median (IQR)	4.5 (3.1 to 6.0)	4.3 (3.0 to 5.9)	4.3 (2.8 to 5.8)	5.0 (3.9 to 6.2)	4.35 (3.10 to 5.93)	3.95 (2.4 to 5.45)	4.7 (2.85 to 6.0)	<0.001 *
Basophils %	Median (IQR)	0.3 (0.2 to 0.5)	0.3 (0.2 to 0.5)	0.3 (0.2 to 0.5)	0.4 (0.3 to 0.7)	0.4 (0.2 to 0.6)	0.3 (0.2 to 0.5)	0.3 (0.2 to 0.5)	<0.001 *
RBC count (million/cumm)	Median (IQR)	4.63 (4.16 to 5.15)	4.68 (4.21 to 5.2)	4.66 (4.28 to 5.21)	4.59 (4.15 to 5.06)	4.70 (4.35 to 5.05)	4.46 (4.0 to 4.97)	4.58 (4.09 to 5.23)	0.002 *
Platelet count (lakh cells/cumm)	Median (IQR)	2.49 (1.94 to 3.17)	2.51 (1.97 to 3.27)	2.46 (1.83 to 3.19)	2.5 (2.08 to 3.03)	2.47 (2.03 to 2.84)	2.43 (1.69 to 3.11)	2.54 (1.97 to 3.29)	0.483 *
NLR	Median (IQR)	5.9 (3.3 to 12.0)	6.55 (3.8 to 13.3)	8.50 (4.6 to 17.6)	3.3 (2.2 to 5.30)	4.05 (2.4 to 7.35)	8.50 (4.7 to 17.8)	5.40 (3.5 to 10.3)	<0.001 *

* Kruskal–Wallis test. ^†^ Pearson Chi sq. test. LOS: Length of hospital stay, AECOPD: Acute exacerbation of chronic obstructive pulmonary diseases, NBE: No bacterial infection and no eosinophilia, B: Bacterial infection, E: Eosinophilia and no bacterial infections, BE: Bacterial infection with eosinophilia, P: Pneumonia, BC: Bronchiectasis, NTS and NBMS: Non-tobacco smoke and non-biomass smoke, TS: Tobacco smoke, BMS: Biomass smoke, TS and BMS: Tobacco smoke and biomass smoke, CCI: Charlson comorbidity index, OSA: Obstructive sleep apnea, PAH: Pulmonary hypertension, T1RF: Type 1 respiratory failure, T2RF: Type 2 respiratory failure, NLR: Neutrophil–lymphocytes ratio.

**Table 2:** Clinical characteristics of AECOPD phenotypes categorized based on exposure type.

		NTS- and NBMS-AECOPD (N = 637)	TS-AECOPD (N = 1100)	BMS-AECOPD (N = 425)	TS- and BMS-AECOPD (N = 32)	*p*-Value
Age in years	Median (IQR)	68 (61.0 to 75.0)	66 (60.0 to 73.0)	66 (58.0 to 74.0)	65 (61.5 to 68.3)	<0.01 *
Male	Yes (n, %)	498 (78.2)	1091 (99.2)	28 (6.6)	32 (100)	<0.01 ^†^
Female	Yes (n, %)	139 (21.8)	9 (0.8)	397 (93.4)	0 (0)	
LOS in days	Median (IQR)	5.0 (3.0 to 8.0)	5.0 (3.0 to 7.0)	6.0 (4.0 to 8.0)	5.0 (3.0 to 7.0)	0.019 *
Ward	Ward (n, %)	516 (81.0)	906 (82.4)	347 (81.6)	25 (78.1)	0.85 ^†^
ICU	ICU (n, %)	121 (19)	194 (17.6)	78 (18.4)	7 (21.9)	
Alive	Alive (n, %)	627 (98.4)	1070 (97.3)	395 (92.9)	30 (93.8)	<0.01 ^†^
Dead	Dead (n, %)	10 (1.6)	30 (2.7)	30 (7.1)	2 (6.3)	
CCI	Median (IQR)	4.0 (3.0 to 5.0)	4.0 (3.0 to 5.0)	4.0 (3.0 to 5.0)	3.0 (3.0 to 4.0)	<0.01 *
Bacterial infection	Yes (n, %)	131 (20.6)	198 (18.0)	92 (21.6)	11 (34.4)	0.05 ^†^
Alcohol consumption	Yes (n, %)	19 (3.0)	283 (25.7)	1 (0.2)	7 (21.9)	<0.01 ^†^
Diabetes mellitus	Yes (n, %)	184 (28.9)	223 (20.3)	110 (25.9)	5 (15.6)	<0.01 ^†^
Heart diseases	Yes (n, %)	134 (21.0)	140 (12.7)	49 (11.5)	4 (12.5)	<0.01 ^†^
Renal diseases	Yes (n, %)	74 (11.6)	87 (7.9)	25 (5.9)	0 (0)	<0.01 ^†^
Liver diseases	Yes (n, %)	19 (3)	32 (2.9)	4 (0.9)	1 (3.1)	0.14 ^†^
Cor pulmonale	Yes (n, %)	141 (22.1)	180 (16.4)	122 (28.7)	9 (28.1)	<0.01 ^†^
Hypertension	Yes (n, %)	268 (42.1)	293 (26.6)	189 (44.5)	8 (25.0)	<0.01 ^†^
Obesity	Yes (n, %)	38 (6.0)	40 (3.6)	49 (11.5)	2 (6.3)	<0.01 ^†^
OSA	Yes (n, %)	39 (6.1)	42 (3.8)	54 (12.7)	2 (6.3)	<0.01 ^†^
PAH	Yes (n, %)	92 (14.4)	195 (17.7)	88 (20.7)	8 (25.0)	0.04 ^†^
Sepsis	Yes (n, %)	26 (4.1)	46 (4.2)	20 (4.7)	2 (6.3)	0.90 ^†^
T1RF	Yes (n, %)	16 (2.5)	47 (4.3)	17 (4.0)	0 (0)	0.18 ^†^
T2RF	Yes (n, %)	100 (15.7)	166 (15.1)	96 (22.6)	9 (28.1)	<0.01 ^†^
AECOPD-NBE	Yes (n, %)	295 (46.3)	560 (50.9)	164 (38.6)	6 (18.8)	<0.01 ^†^
AECOPD-E	Yes (n, %)	128 (20.1)	179 (16.3)	77 (18.1)	4 (12.5)	
AECOPD-P	Yes (n, %)	76 (11.9)	165 (15.0)	66 (15.5)	2 (6.3)	
AECOPD-BC	Yes (n, %)	46 (7.2)	61 (5.5)	51 (12.0)	10 (31.3)	
AECOPD-B	Yes (n, %)	75 (11.8)	114 (10.4)	54 (12.7)	8 (25.0)	
AECOPD-BE	Yes (n, %)	17 (2.7)	21 (1.9)	13 (3.1)	2 (6.3)	
Lymphocytes %	Median (IQR)	13.5 (7.45 to 22.1)	12.9 (6.8 to 20)	15.25 (9.6 to 22.1)	12.8 (6.6 to 23.9)	<0.001 *
Eosinophils %	Median (IQR)	1.3 (0.2 to 3.6)	0.85 (0.1 to 3.0)	1.3 (0.2 to 3.6)	2.4 (0.4 to 3.9)	0.001 *
Monocytes %	Median (IQR)	4.6 (3.3 to 6.2)	4.5 (3.0 to 6.0)	4.2 (3.1 to 5.4)	5.2 (3.2 to 6.4)	0.068 *
Basophils %	Median (IQR)	0.3 (0.2 to 0.5)	0.3 (0.2 to 0.5)	0.3 (0.2 to 0.5)	0.3 (0.2 to 0.5)	0.811 *
RBC count (million/cumm)	Median (IQR)	4.5 (4.0 to 5.1)	4.7 (4.3 to 5.3)	4.46 (4.1 to 4.9)	4.83 (4.6 to 5.3)	<0.001 *
Platelet count (lakh cells/cumm)	Median (IQR)	2.47 (1.91 to 3.1)	2.46 (1.86 to 3.18)	2.65 (2.16 to 3.32)	2.090 (1.95 to 2.5)	< 0.001 *
NLR	Median (IQR)	5.90 (3.0 to 11.6)	6.10 (3.5 to 13.2)	5.15 (3.2 to 8.77)	6.10 (3.0 to 13.4)	0.002 *

* Kruskal–Wallis test. ^†^ Pearson Chi sq. test. LOS: Length of hospital stay, AECOPD: Acute exacerbation of chronic obstructive pulmonary diseases, NBE: No bacterial infection and no eosinophilia, B: Bacterial infection, E: Eosinophilia and no bacterial infections, BE: Bacterial infection with eosinophilia, P: Pneumonia, BC: Bronchiectasis, NTS and NBMS: Non-tobacco smoke and non-biomass smoke, TS: Tobacco smoke, BMS: Biomass smoke, TS and BMS: Tobacco smoke and biomass smoke, CCI: Charlson comorbidity index, OSA: Obstructive sleep apnea, PAH: Pulmonary hypertension, T1RF: Type 1 respiratory failure, T2RF: Type 2 respiratory failure, NLR: Neutrophil–lymphocytes ratio.

**Table 3:** Pairwise comparisons for AECOPD phenotypes categorized based on pathology.

Levels	Levels	*p*-Value
AECOPD-E	AECOPD-NBE	0.529
AECOPD-P	AECOPD-NBE	<0.001
AECOPD-P	AECOPD-E	<0.001
AECOPD-BC	AECOPD-NBE	0.146
AECOPD-BC	AECOPD-E	0.008
AECOPD-BC	AECOPD-P	1.000
AECOPD-B	AECOPD-NBE	<0.001
AECOPD-B	AECOPD-E	<0.001
AECOPD-B	AECOPD-P	1.000
AECOPD-B	AECOPD-BC	0.389
AECOPD-BE	AECOPD-NBE	0.356
AECOPD-BE	AECOPD-E	0.090
AECOPD-BE	AECOPD-P	1.000
AECOPD-BE	AECOPD-BC	1.000
AECOPD-BE	AECOPD-B	1.000

Note: *p*-value adjusted using Holm method. AECOPD: Acute exacerbation of chronic obstructive pulmonary diseases, NBE: No bacterial infection and no eosinophilia, B: Bacterial infection, E: Eosinophilia and no bacterial infections, BE: Bacterial infection with eosinophilia, P: Pneumonia, and BC: Bronchiectasis.

**Table 4:** Pairwise comparisons of AECOPD phenotypes categorized based on exposure type.

Levels	Levels	*p*-Value
TS-AECOPD	NTS and NBMS-AECOPD	0.095
BMS-AECOPD	NTS and NBMS-AECOPD	<0.001
BMS-AECOPD	TS-AECOPD	0.009
TS and BMS-AECOPD	NTS and NBMS-AECOPD	0.034
TS and BMS-AECOPD	TS-AECOPD	0.210
TS and BMS-AECOPD	BMS-AECOPD	0.838

Note. *p*-value adjusted using the Holm method. AECOPD: Acute exacerbation of chronic obstructive pulmonary diseases, NTS and NBMS: Non-tobacco smoke and non-biomass smoke, TS: Tobacco smoke, BMS: Biomass smoke, and TS and BMS: Tobacco smoke and biomass smoke.

**Table 5:** Hazard ratio (H.R.) reflecting mortality risk was calculated by multivariate Cox regression analysis.

		Model 1: Pathology		Model 2: Exposure	
		HR (Univariable)	HR (Multivariable)	HR (Univariable)	HR (Multivariable)
Sex	Male	Reference	Reference	**-**	**-**
	Female	2.47 (1.55–3.93) ***	3.05 (1.83–5.09) ***	**-**	**-**
Age	Mean (SD)	1.02 (1.00–1.05) *	1.03 (1.00–1.06)	1.02 (1.00–1.05) *	1.03 (1.00–1.06)
Cor pulmonale	Yes	1.37 (0.83–2.26)	0.76 (0.44–1.32)	1.37 (0.83–2.26)	0.91 (0.54–1.54)
T1RF	Yes	0.83 (0.20–3.40)	0.88 (0.21–3.66)	0.83 (0.20–3.40)	1.01 (0.24–4.21)
T2RF	Yes	2.21 (1.37–3.56) ***	1.74 (1.04–2.91) *	2.21 (1.37–3.56) ***	1.75 (1.04–2.93) *
Hypertension	Yes	1.00 (0.62–1.62)	0.70 (0.41–1.20)	1.00 (0.62–1.62)	0.80 (0.47–1.34)
PAH	Yes	1.07 (0.60–1.92)	0.86 (0.46–1.58)	1.07 (0.60–1.92)	0.85 (0.47–1.55)
Sepsis	Yes	5.65 (3.29–9.72) ***	3.13 (1.72–5.71) ***	5.65 (3.29–9.72) ***	5.23 (2.96–9.25) ***
CCI	Mean (SD)	1.10 (0.95–1.27)	1.07 (0.91–1.26)	1.10 (0.95–1.27)	0.97 (0.78–1.22)
AECOPD phenotypes based on Exposure type	NTS and NBMS-AECOPD	**-**	**-**	Reference	Reference
	TS-AECOPD	**-**	**-**	2.18 (1.04–4.61) *	2.17 (1.02–4.63) *
	BMS-AECOPD	**-**	**-**	4.82 (2.29–10.16) ***	5.28 (2.46–11.35) ***
	TS and BMS-AECOPD	**-**	**-**	6.35 (1.36–29.51) *	7.24 (1.53–34.29) *
AECOPD phenotypes based on pathology	AECOPD-NBE	Reference	Reference	**-**	**-**
	AECOPD-E	0.00 (0.00-Inf)	0.00 (0.00-Inf)	**-**	**-**
	AECOPD-B	7.21 (3.47–14.97) ***	6.42 (3.06–13.46) ***	**-**	**-**
	AECOPD-BE	3.53 (0.96–12.89)	3.84 (1.04–14.20) *	**-**	**-**
	AECOPD-P	5.34 (2.54–11.20) ***	4.33 (2.01–9.30) ***	**-**	**-**
	AECOPD-BC	3.30 (1.25–8.72) *	2.72 (1.00–7.38) *	**-**	**-**

* = *p* < 0.05, ** = *p* < 0.01, *** = *p* < 0.001, AECOPD: Acute exacerbation of chronic obstructive pulmonary diseases, NBE: No bacterial infection and no eosinophilia, B: Bacterial infection, E: Eosinophilia and no bacterial infections, BE: Bacterial infection with eosinophilia, P: Pneumonia, BC: Bronchiectasis, NTS and NBMS: Non-tobacco smoke and non-biomass smoke, TS: Tobacco smoke, BMS: Biomass smoke, TS and BMS: Tobacco smoke and biomass smoke, CCI: Charlson comorbidity index, PAH: Pulmonary hypertension, T1RF: Type 1 respiratory failure, T2RF: Type 2 respiratory failure. The exposure is already segregated by gender: 99.2% of TS-AECOPD subjects are males and 93.4% of BMS-AECOPD subjects are females; therefore, gender was not used in model 2.

## Data Availability

All data generated or analyzed during this study are included in this published article and are available from the corresponding author upon reasonable request.

## Supplementary Materials

The following supporting information can be downloaded at: https://www.mdpi.com/article/10.3390/toxics10110667/s1, Table S1: Clinical characteristics of AECOPD subjects based on gender; Table S2: Clinical Characteristics of AECOPD subjects based on pathology with gender stratified; Table S3: Clinical Characteristics of AECOPD subjects based on exposure type with gender stratified; Table S4: Binomial logistic regression for the type of ward as an outcome; Table S5: Binomial logistic regression for the length of hospital stays as an outcome.
