# Supplementary material for: Impact of Acute Exacerbation and Its Phenotypes on the Clinical Outcomes of Chronic Obstructive Pulmonary Disease in Hospitalized Patients: A Cross-Sectional Study
Source: Toxics. 2022 Nov 6;10(11):667. doi: 10.3390/toxics10110667 (PMC9695923; doi:10.3390/toxics10110667)
Supplement: Supplementary file 1 [file toxics-10-00667-s001.zip › toxics-1970437-supplementary.pdf]

Article

Impact of Acute Exacerbation and its Phenotypes on the Clinical Outcomes of Chronic Obstructive Pulmonary Disease in Hospitalized Patients: Supplementary Material

Mohammed Kaleem Ullah [1,2] , Ashwaghosha Parthasarathi [3,4], Jayaraj Biligere Siddaiah [5], Prashant Vishwanath [1], Swapna Upadhyay [6], Koustav Ganguly [6,*,†] and Padukudru Anand Mahesh [5,*,†]

1 Centre for Excellence in Molecular Biology and Regenerative Medicine, Department of Biochemistry, JSS Medical College, JSSAHER, Mysore 570015, Karnataka, India
2 Global Infectious Diseases Fellow, Division of Infectious Disease and Vaccinology, School of Public Health, University of California, Berkeley, CA 94720, USA
3 Allergy, Asthma, and Chest Centre, Krishnamurthypuram, Mysore 570004, Karnataka, India
4 RUTGERS Centre for Pharmacoepidemiology and Treatment Science, New Brunswick, NJ 08901-1293, USA
5 Department of Respiratory Medicine, JSS Medical College, JSSAHER, Mysore 570015, Karnataka, India
6 Unit of Integrative Toxicology, Institute of Environmental Medicine (IMM), Karolinska Institutet, 17177 Stockholm, Sweden
* Correspondence: koustav.ganguly@ki.se (K.G.); pamahesh@jssuni.edu.in (P.A.M.)
† These authors contributed equally to this work.

Table S1: Clinical characteristics of AECOPD subjects based on gender

		Male (N=1649)	Female (N=545)	p-value
Age in years	Median (IQR)	67 (60.0 to 74.0)	66 (60.0 to 75.0)	0.76*
LOS in days	Median (IQR)	5 (3.0 to 7.00)	6 (4.0 to 8.00)	0.006*
Ward	Ward (n, %)	1347 (81.7)	447 (82.0)	0.86†
ICU	ICU (n, %)	302 (18.3)	98 (18.0)	
Alive	Alive (n, %)	1611 (97.7)	511 (93.8)	<0.001†
Dead	Dead (n, %)	38 (2.3)	34 (6.2)	
Bacterial infection	Yes (n, %)	330 (20.0)	102 (18.7)	0.51†
Alcohol consumption	Yes (n, %)	307 (18.6)	3 (0.6)	<0.001†
NTS & NBMS-AECOPD	Yes (n, %)	498 (30.2)	139 (25.5)	<0.001†
TS-AECOPD	Yes (n, %)	1091 (66.2)	9 (1.7)	
BMS-AECOPD	Yes (n, %)	28 (1.7)	397 (72.8)	
TS & BMS-AECOPD	Yes (n, %)	32 (1.9)	0 (0)	
CCI	Median (IQR)	4 (3.0 to 5.00)	4 (3.0 to 5.00)	0.011*
Diabetes mellitus	Yes (n, %)	355 (21.5)	167 (30.6)	<0.01†
Heart diseases	Yes (n, %)	258 (15.6)	69 (12.7)	0.09†
Renal diseases	Yes (n, %)	155 (9.4)	31 (5.7)	0.007†
Liver diseases	Yes (n, %)	53 (3.2)	3 (0.6)	<0.001†
Corpulmonale	Yes (n, %)	288 (17.5)	164 (30.1)	<0.001†
Hypertension	Yes (n, %)	485 (29.4)	273 (50.1)	<0.001†
Obesity	Yes (n, %)	54(3.3)	75 (13.8)	<0.001†
OSA	Yes (n, %)	53 (3.2)	84 (15.4)	<0.001†
PAH	Yes (n, %)	257 (15.6)	126 (23.1)	<0.001†
Sepsis	Yes (n, %)	73 (4.4)	21 (3.9)	0.57†
T1RF	Yes (n, %)	57 (3.5)	23 (4.2)	0.41†
T2RF	Yes (n, %)	245 (14.9)	126 (23.1)	<0.001†
AE COPD-NBE	Yes (n, %)	805 (48.8)	220 (40.4)	<0.001†
AECOPD-E	Yes (n, %)	279 (16.9)	109 (20.0)	
AECOPD-P	Yes (n, %)	230 (13.9)	79 (14.5)	
AECOPD-BC	Yes (n, %)	106 (6.4)	62 (11.4)	
AECOPD-B	Yes (n, %)	189 (11.5)	62 (11.4)	
AECOPD-BE	Yes (n, %)	40 (2.4)	13 (2.4)	
Lymphocytes %	Median (IQR)	12.95 (6.9 to 20.60)	14.95 (9.1 to 22.20)	< .001*

Eosinophils %	Median (IQR)	1.0 (0.1 to 3.20)	1.3 (0.2 to 3.60)	0.071*
Monocytes %	Median (IQR)	4.6 (3.1 to 6.20)	4.2 (3.0 to 5.40)	< .001*
Basophils %	Median (IQR)	0.3 (0.2 to 0.50)	0.3 (0.2 to 0.50)	0.882*
RBC count (million/cumm)	Median (IQR)	4.68 (4.2 to 5.24)	4.43 (4.06 to 4.91)	< .001*
Platelet count (lakh cells/cumm)	Median (IQR)	2.43 (1.87 to 3.08)	2.74 (2.22 to 3.41)	< .001*
Neutrophil-lymphocytes ratio (NLR)	Median (IQR)	6.1 (3.40 to 12.75)	5.2 (3.20 to 9.32)	0.002*

*Kruskal-Wallis test. †Pearson Chi sq test.

LOS-Length of hospital stay, AECOPD=Acute exacerbation of chronic obstructive pulmonary diseases, NBE=No bacterial and no eosinophilia, B-Bacterial, E-Eosinophilia, and no bacterial infections, BE- bacterial infection with eosinophilia, P-pneumonia, and BC-bronchiectasis, NTS & NBMS: Non-tobacco smoke and non-biomass smoke, TS: Tobacco smoke, BMS: Biomass smoke, TS & BMS: Tobacco smoke and biomass smoke, CCI- Charlson comorbidity index, OSA-Obstructive sleep apnea, PAH-pulmonary hypertension, T1RF-Type 1 respiratory failure, T2RF-Type 2 respiratory failure, NLR-Neutrophil-lymphocytes ratio

Table S2: Clinical Characteristics of AECOPD subjects based on pathology with gender stratified

		AECOPD-NBE (N=1025) M (n=805)	F (n=220)	p-Value	AECOPD-B (N=251) M (n=189)	F (n=62)	p-Value	AECOPD-E (N=388) M (n=279)	F (n=109)	p-Value	AECOPD-BE (N=53) M (n=40)	F (n=13)	p-Value	AECOPD-P (N=309) M (n=230)	F (n=79)	p-Value	AECOPD-BC (N=168) M (n=106)	F (n=62)	p-Value
Age in years	Median (IQR)	67.0 (60.0 to 74.0)	67.0 (60.0 to 75.0)	0.680*	66.0 (60.0 to 74.0)	68.5 (60.3 to 74.8)	0.357*	66.0 (60.0 to 73.0)	66.0 (58.0 to 74.0)	0.956*	70.0 (59.8 to 75.3)	70.0 (57.0 to 73.0)	0.610*	66.5 (59.0 to 75.0)	67.0 (60.0 to 76.5)	0.557*	65.0 (57.3 to 70.0)	63.0 (54.5 to 73.8)	0.928*
LOS in days	Median (IQR)	5.0 (3.0 to 7.0)	5.0 (4.0 to 8.0)	< 0.001*	6.0 (4.0 to 9.0)	7.0 (4.0 to 10.0)	0.671*	4.0 (3.0 to 6.0)	5.0 (3.0 to 7.0)	0.016*	5.5 (4.0 to 9.0)	7.0 (5.0 to 9.0)	0.972*	6.0 (4.0 to 10.8)	6.0 (4.0 to 9.0)	0.223*	5.0 (3.0 to 8.0)	6.0 (4.0 to 9.0)	0.200*
Admission to Ward /ICU	Ward (n, %)	724.0 (89.9)	202.0 (91.8)	0.403†	93.0 (49.2)	30.0 (48.4)	0.911†	273.0 (97.8)	107.0 (98.2)	0.844†	31.0 (77.5)	6.0 (46.2)	0.032†	147.0 (63.9)	52.0 (65.8)	0.760†	79.0 (74.5)	50.0 (80.6)	0.3665†
	ICU (n, %)	81.0 (10.1)	18.0 (8.2)		96.0 (50.8)	32.0 (51.6)		6.0 (2.2)	2.0 (1.8)		9.0 (22.5)	7.0 (53.8)		83.0 (36.1)	27.0 (34.2)		27.0 (25.5)	12.0 (19.4)	
Hospital outcome	Alive (n, %)	801.0 (99.5)	214.0 (97.3)	0.003†	174.0 (92.1)	49.0 (79.0)	0.005†	279.0 (100.0)	109.0 (100.0)	-	39.0 (97.5)	11.0 (84.6)	0.081†	215.0 (93.5)	70.0 (88.6)	0.163†	103.0 (97.2)	58.0 (93.5)	0.257†
	Dead (n, %)	4.0 (0.5)	6.0 (2.7)		15.0 (7.9)	13.0 (21.0)		0	0		1.0 (2.5)	2.0 (15.4)		15.0 (6.5)	9.0 (11.4)		3.0 (2.8)	4.0 (6.5)	
Bacterial infection	Yes (n, %)	0	0	-	189.0 (100.0)	62.0 (100.0)	< 0.001†	0	0	-	40.0 (100.0)	13.0 (100.0)	< 0.001†	71.0 (30.9)	14.0 (17.7)	0.024†	30.0 (28.3)	13.0 (21.0)	0.293†
Alcohol consumption	Yes (n, %)	169.0 (21.0)	1.0 (0.5)	<0.001†	20.0 (10.6)	1.0 (1.6)	0.027†	52.0 (18.6)	0.0 (0.0)	<0.001†	2.0 (5.0)	0.0 (0.0)	0.411†	50.0 (21.7)	0.0 (0.0)	<0.001†	14.0 (13.2)	1.0 (1.6)	0.011†
NTS & NBMS-AECOPD	Yes (n, %)	236.0 (29.3)	59.0 (26.8)	<0.001†	62.0 (32.8)	13.0 (21.0)	<0.001†	93.0 (33.3)	35.0 (32.1)	<0.001†	15.0 (37.5)	2.0 (15.4)	<0.001†	60.0 (26.1)	16.0 (20.3)	<0.001†	32.0 (30.2)	14.0 (22.6)	<0.001†

TS-AECOPD	Yes (n, %)	557.0 (69.2)	3.0 (1.4)		113.0 (59.8)	1.0 (1.6)		177.0 (63.4)	2.0 (1.8)		21.0 (52.5)	0.0 (0.0)		165.0 (71.7)	0.0 (0.0)		58.0 (54.7)	3.0 (4.8)	
BMS-AECOPD	Yes (n, %)	6.0 (0.7)	158.0 (71.8)		6.0 (3.2)	48.0 (77.4)		5.0 (1.8)	72.0 (66.1)		2.0 (5.0)	11.0 (84.6)		3.0 (1.3)	63.0 (79.7)		6.0 (5.7)	45.0 (72.6)	
TS & BMS-AECOPD	Yes (n, %)	6.0 (0.7)	0.0 (0.0)		8.0 (4.2)	0.0 (0.0)		4.0 (1.4)	0.0 (0.0)		2.0 (5.0)	0.0 (0.0)		2.0 (0.9)	0.0 (0.0)		10.0 (9.4)	0.0 (0.0)	
CCI	Median (IQR)	4.0 (3.0 to 5.0)	4.0 (3.0 to 5.0)	0.976*	4.0 (3.0 to 5.0)	4.0 (3.0 to 5.0)	0.881*	4.0 (3.0 to 5.0)	3.0 (2.0 to 4.0)	0.001*	4.0 (3.0 to 5.0)	4.0 (3.0 to 5.0)	0.800*	4.0 (3.0 to 5.0)	4.0 (3.0 to 5.0)	0.281*	3.0 (3.0 to 4.0)	3.0 (2.0 to 4.0)	0.644*
Comorbidities																			
Diabetes mellitus	Yes (n, %)	171.0 (21.2)	78.0 (35.5)	<0.001†	42.0 (22.2)	18.0 (29.0)	0.275†	57.0 (20.4)	31.0 (28.4)	0.090†	8.0 (20.0)	6.0 (46.2)	0.063†	58.0 (25.2)	19.0 (24.1)	0.836†	19.0 (17.9)	15.0 (24.2)	0.329†
Heart diseases	Yes (n, %)	130.0 (16.1)	32.0 (14.5)	0.563†	31.0 (16.4)	6.0 (9.7)	0.195†	43.0 (15.4)	10.0 (9.2)	0.108†	8.0 (20.0)	1.0 (7.7)	0.305†	32.0 (13.9)	13.0 (16.5)	0.580†	14.0 (13.2)	7.0 (11.3)	0.717†
Renal diseases	Yes (n, %)	71.0 (8.8)	15.0 (6.8)	0.343†	21.0 (11.1)	5.0 (8.1)	0.495†	16.0 (5.7)	4.0 (3.7)	0.408†	5.0 (12.5)	0.0 (0.0)	0.180†	36.0 (15.7)	5.0 (6.3)	0.035†	6.0 (5.7)	2.0 (3.2)	0.475†
Liver diseases	Yes (n, %)	28.0 (3.5)	1.0 (0.5)	0.017†	13.0 (6.9)	0.0 (0.0)	0.034†	4.0 (1.4)	1.0 (0.9)	0.685†	0.0 (0.0)	1.0 (7.7)	0.077†	7.0 (3.0)	0.0 (0.0)	0.117†	1.0 (0.9)	0.0 (0.0)	0.443†
Corpulmonale	Yes (n, %)	134.0 (16.6)	83.0 (37.7)	<0.001†	38.0 (20.1)	24.0 (38.7)	0.003†	38.0 (13.6)	19.0 (17.4)	0.341†	2.0 (5.0)	3.0 (23.1)	0.053†	43.0 (18.7)	20.0 (25.3)	0.208†	33.0 (31.1)	15.0 (24.2)	0.337†
Hypertension	Yes (n, %)	242.0 (30.1)	120.0 (54.5)	<0.001†	56.0 (29.6)	32.0 (51.6)	0.002†	80.0 (28.7)	60.0 (55.0)	<0.001†	16.0 (40.0)	7.0 (53.8)	0.382†	60.0 (26.1)	40.0 (50.6)	<0.001†	31.0 (29.2)	14.0 (22.6)	0.347†
Obesity	Yes (n, %)	27.0 (3.4)	34.0 (15.5)	<0.001†	6.0 (3.2)	8.0 (12.9)	0.004†	11.0 (3.9)	22.0 (20.2)	<0.001†	5.0 (12.5)	2.0 (15.4)	0.790†	4.0 (1.7)	8.0 (10.1)	<0.001†	1.0 (0.9)	1.0 (1.6)	0.699†
OSA	Yes (n, %)	25.0 (3.1)	39.0 (17.7)	<0.001†	5.0 (2.6)	7.0 (11.3)	0.006†	13.0 (4.7)	24.0 (22.0)	<0.001†	4.0 (10.0)	3.0 (23.1)	0.226†	6.0 (2.6)	10.0 (12.7)	<0.001†	0.0 (0.0)	1.0 (1.6)	0.190†
PAH	Yes (n, %)	117.0 (14.5)	46.0 (20.9)	0.022†	38.0 (20.1)	11.0 (17.7)	0.684†	37.0 (13.3)	28.0 (25.7)	0.002†	11.0 (27.5)	6.0 (46.2)	0.211†	30.0 (13.0)	16.0 (20.3)	0.120†	24.0 (22.6)	19.0 (30.6)	0.251†

Sepsis	Yes (n, %)	14.0 (1.7)	5.0 (2.3)	0.603†	10.0 (5.3)	5.0 (8.1)	0.424†	1.0 (0.4)	0.0 (0.0)	0.531†	0	0	-	42.0 (18.3)	11.0 (13.9)	0.378†	6.0 (5.7)	0.0 (0.0)	0.056†
T1RF	Yes (n, %)	27.0 (3.4)	8.0 (3.6)	0.838†	7.0 (3.7)	5.0 (8.1)	0.163†	6.0 (2.2)	7.0 (6.4)	0.036†	1.0 (2.5)	0.0 (0.0)	0.565†	13.0 (5.7)	2.0 (2.5)	0.266†	3.0 (2.8)	1.0 (1.6)	0.618†
T2RF	Yes (n, %)	115.0 (14.3)	51.0 (23.2)	0.002†	42.0 (22.2)	19.0 (30.6)	0.180†	16.0 (5.7)	14.0 (12.8)	0.018†	8.0 (20.0)	6.0 (46.2)	0.063†	45.0 (19.6)	24.0 (30.4)	0.046†	19.0 (17.9)	12.0 (19.4)	0.818†
Lymphocytes %	Median (IQR)	12.0 (6.5 to 18.6)	14.4 (9.0 to 21.3)	0.002*	9.7 (4.8 to 15.9)	11.0 (6.0 to 20.2)	0.057*	20.6 (14.6 to 27.5)	19.9 (13.3 to 27.4)	0.671*	18.1 (11.3 to 25.3)	18.1 (10.6 to 25.6)	0.612*	9.0 (4.8 to 16.0)	12.2 (7.2 to 17.5)	0.168*	12.3 (7.2 to 19.7)	16.4 (11.1 to 20.2)	0.065*
Eosinophils %	Median (IQR)	0.5 (0.1 to 1.3)	0.5 (0.1 to 1.3)	0.595*	0.3 (0.0 to 1.2)	0.2 (0.1 to 1.4)	0.545*	5.0 (3.9 to 7.0)	4.6 (3.6 to 6.0)	0.006*	4.5 (3.8 to 5.7)	4.2 (3.8 to 4.6)	0.271*	0.4 (0.1 to 1.6)	0.9 (0.1 to 2.4)	0.115*	0.8 (0.4 to 3.0)	0.9 (0.1 to 2.9)	0.361*
Monocytes %	Median (IQR)	4.3 (3.0 to 6.0)	4.3 (3.1 to 5.6)	0.166*	4.3 (2.8 to 5.8)	4.3 (3.0 to 5.8)	0.961*	5.2 (4.2 to 6.4)	4.3 (3.2 to 5.1)	< 0.001*	4.5 (3.1 to 6.3)	4.2 (3.3 to 5.3)	0.699*	4.0 (2.3 to 6.2)	3.8 (2.8 to 4.8)	0.154*	4.9 (2.9 to 6.6)	4.2 (2.8 to 5.2)	0.757*
Basophils %	Median (IQR)	0.3 (0.2 to 0.4)	0.3 (0.2 to 0.5)	0.520*	0.3 (0.2 to 0.4)	0.3 (0.2 to 0.5)	0.924*	0.5 (0.3 to 0.7)	0.4 (0.2 to 0.6)	0.023*	0.4 (0.3 to 0.6)	0.3 (0.2 to 0.5)	0.161*	0.3 (0.2 to 0.5)	0.3 (0.2 to 0.5)	0.302*	0.3 (0.2 to 0.5)	0.3 (0.2 to 0.4)	0.163*
RBC count (million/cumm)	Median (IQR)	4.7 (4.3 to 5.3)	4.4 (4.0 to 5.1)	0.061*	4.7 (4.3 to 5.2)	4.6 (4.2 to 5.0)	0.527*	4.7 (4.2 to 5.2)	4.4 (4.1 to 4.7)	0.005*	4.7 (4.4 to 5.1)	4.6 (4.3 to 5.1)	0.490*	4.5 (4.0 to 5.0)	4.3 (3.8 to 4.9)	0.146*	4.8 (4.3 to 5.4)	4.4 (3.9 to 4.8)	< 0.001*
Platelet count (lakh cells/cumm)	Median (IQR)	2.5 (1.9 to 3.2)	2.8 (2.2 to 3.6)	< 0.001*	2.3 (1.8 to 3.1)	2.9 (2.2 to 3.6)	0.040*	2.5 (2.0 to 3.0)	2.7 (2.3 to 3.0)	0.007*	2.4 (2.0 to 2.8)	2.5 (2.3 to 2.8)	0.904*	2.3 (1.6 to 3.1)	2.5 (2.0 to 3.0)	0.254*	2.2 (1.9 to 3.0)	2.8 (2.6 to 3.6)	< 0.001*
Neutrophil-lymphocytes ratio (NLR)	Median (IQR)	6.8 (4.0 to 14.0)	5.6 (3.4 to 9.7)	0.015*	8.8 (4.9 to 18.8)	7.6 (3.5 to 15.0)	0.374*	3.3 (2.1 to 5.1)	3.5 (2.2 to 6.2)	0.091*	4.1 (2.4 to 6.8)	4.0 (2.5 to 8.1)	0.657*	9.7 (4.9 to 18.9)	6.8 (4.4 to 12.3)	0.064*	6.4 (3.6 to 12.2)	4.8 (3.3 to 7.6)	0.426*

*Kruskal-Wallis test. †Pearson Chi sq test.

LOS-Length of hospital stay, AECOPD=Acute exacerbation of chronic obstructive pulmonary diseases, NBE=No bacterial and no eosinophilia, B-Bacterial, E-Eosinophilia, and no bacterial infections, BE-bacterial infection with eosinophilia, P-pneumonia, and BC-bronchiectasis, NTS & NBMS: Non-tobacco smoke and non-biomass smoke, TS: Tobacco smoke, BMS: Biomass smoke, TS & BMS: Tobacco smoke and biomass smoke, CCI- Charlson comorbidity index, OSA-Obstructive sleep apnea, PAH-pulmonary hypertension, T1RF-Type 1 respiratory failure, T2RF-Type 2 respiratory failure, NLR-Neutrophil-lymphocytes ratio

Table S3: Clinical Characteristics of AECOPD subjects based on exposure type with gender stratified

		NTS & NBMS-AECOPD (N=637)			TS-AECOPD (N=1100)			BMS-AECOPD (N=425)			TS & BMS-AECOPD (N=32)	
		**Male (n=498)**	**Female (n=139)**	**p-value**	**Male (n=1091)**	**Female (n=9)**	**p-value**	**Male (n=28)**	**Female (n=397)**	**p-value**	**Male (n=32)**	**Fe-male (n=0)**
Age in years	Median (IQR)	68.0 (61.0 to 75.0)	68 (62.0 to76.0)	0.288*	66.0 (60.0 to73.0)	67 (65.0 to 68.0)	0.759*	67.5 (58.25 to70.25)	66 (58.0 to 74.0)	0.630*	65.0 (61.5 to 68.25)	0
LOS in days	Median (IQR)	5.0 (3.0 to 7.0)	6 (4.0 to 9.0)	0.149*	5 (3.0 to 7.0)	4 (4.0 to 7.0)	0.677*	6.0 (4.0 to 8.0)	5 (4.0 to 8.0)	0.706*	5.0 (3.0 to 7.0)	0
Ward	Ward (n, %)	400.0 (80.3)	116.0 (83.5)	0.405†	899 (82.4)	7 (77.8)	0.717†	23 (82.1)	324 (81.6)	0.944†	25(78.1)	0
ICU	ICU (n, %)	98.0 (19.7)	23.0 (16.5)		192 (17.6)	2 (22.2)		5 (17.9)	73 (18.4)			0
Alive	Alive (n, %)	493.0 (99.0)	134.0 (96.4)	0.030†	1061(97.3)	9 (100.0)	0.614†	27 (96.4)	368 (92.7)	0.456†	30 (93.8)	0
Death	Dead (n, %)	5.0 (1.0)	5.0 (3.6)		30 (2.7)	0 (0)		1 (3.6)	29 (7.3)		2(6.3)	0
CCI	Median (IQR)	4.0 (3.0 to 5.0)	4 (3.0 to 5.0)	0.442*	4 (3.0 to 5.0)	3 (2.0 to 4.0)	0.497*	3.5 (2.0 to 4.0)	4 (3.0 to 5.0)	0.808*	3.0 (3.0 to 4.0)	0
Bacterial infection	Yes (n, %)	111.0 (22.3)	20.0 (14.4)	0.042†	197 (18.1)	1 (11.1)	0.589†	11 (39.3)	81 (20.4)	0.019†	11 (34.4)	0
Alcohol consumption	Yes (n, %)	19.0 (3.8)	0.0 (0.0)	0.019†	281 (25.8)	2 (22.2)	0.809†	0 (0.0)	1 (0.3)	0.790†	7 (21.9)	0
Diabetes mellitus	Yes (n, %)	125.0 (25.1)	59.0 (42.4)	<0.001†	220 (20.2)	3 (33.3)	0.328†	5 (17.9)	105 (26.4)	0.316†	5 (15.6)	0
Heart diseases	Yes (n, %)	108.0 (21.7)	26.0 (18.7)	0.446†	139 (12.7)	1 (11.1)	0.884†	7 (25)	42 (10.6)	0.021†	4 (12.5)	0
Renal diseases	Yes (n, %)	66.0 (13.3)	8.0 (5.8)	0.015†	86 (7.9)	1 (11.1)	0.721†	3 (10.7)	22 (5.5)	0.261†	0 (0)	0
Liver diseases	Yes (n, %)	19.0 (3.8)	0.0 (0.0)	0.019†	32 (2.9)	0 (0)	0.602†	1 (3.6)	3 (0.8)	0.136†	1 (3.1)	0
Corpulmonale	Yes (n, %)	91.0 (18.3)	50.0 (36.0)	<0.001†	179 (16.4)	1 (11.1)	0.669†	9 (32.1)	113 (28.5)	0.677†	9 (28.1)	0
Hypertension	Yes (n, %)	181.0 (36.3)	87.0 (62.6)	<0.001†	288 (26.4)	5 (55.6)	0.049†	8 (28.6)	181 (45.6)	0.080†	8 (25.0)	0
Obesity	Yes (n, %)	12.0 (2.4)	26.0 (18.7)	<0.001†	38 (3.5)	2 (22.2)	0.003†	2 (7.1)	47 (11.8)	0.452†	2 (6.3)	0
OSA	Yes (n, %)	10.0 (2.0)	29.0 (20.9)	<0.001†	39 (3.6)	3 (33.3)	<0.001†	2 (7.1)	52 (13.1)	0.360†	2 (6.3)	0
PAH	Yes (n, %)	51.0 (10.2)	41.0 (29.5)	<0.001†	194 (17.8)	1 (11.1)	0.602†	4 (14.3)	84 (21.2)	0.386†	8 (25.0)	0
Sepsis	Yes (n, %)	23.0 (4.6)	3.0 (2.2)	0.195†	46 (4.2)	0 (0)	0.529†	2 (7.1)	18 (4.5)	0.529†	2 (6.3)	0
T1RF	Yes (n, %)	10.0 (2.0)	6.0 (4.3)	0.124†	47 (4.3)	0 (0)	0.525†	0 (0)	17 (4.3)	0.264†	0 (0)	0
T2RF	Yes (n, %)	66.0 (13.3)	34.0 (24.5)	0.001†	164 (15.0)	2 (22.2	0.548†	6 (21.4)	90 (22.7)	0.879†	9 (28.1)	0
AECOPD-NBE	Yes (n, %)	236 (47.4)	59 (42.4)		557 (51.1)	3 (33.3)		6 (21.4)	158 (39.8)		6 (18.8)	0
AECOPD-E	Yes (n, %)	93 (18.7)	35 (25.2)		177 (16.2)	2 (22.2)		5 (17.9)	72 (18.1)		4 (12.5)	0
AECOPD-P	Yes (n, %)	60 (12.0)	16 (11.5)	0.237†	165 (15.1)	0 (0)	0.011†	3 (10.7)	63 (15.9)	0.136†	2 (6.3)	0
AECOPD-BC	Yes (n, %)	32 (6.4)	14 (10.1)		58 (5.3)	3 (33.3)		6 (21.4)	45 (11.3)		10 (31.3)	0
AECOPD-B	Yes (n, %)	62 (12.4)	13 (9.4)		113 (10.4)	1 (11.1)		6 (21.4)	48 (12.1)		8 (25.0)	0

AECOPD-BE	Yes (n, %)	15 (3.0)	2 (1.4)		21 (1.9)	0 (0)		2 (7.1)	11 (2.8)		2 (6.3)	0
Lymphocytes %	Median (IQR)	13.5 (7.5 to 22.0)	13.55 (7.42 to 21.87)	0.856*	12.9 (6.8 to 19.9)	13.45 (8.3 to 23.4)	0.834*	14.05 (8.425 to 20.37)	15.25 (9.72 to 22.20)	0.718[1]*	12.8 (6.6 to 23.9)	0
Eosinophils %	Median (IQR)	1.3 (0.20 to 3.3)	1.65 (0.30 to 3.90)	0.805*	0.85 (0.10 to 3.0)	1.45 (0.07 to 3.07)	0.679*	1.55 (0.55 to 5.82)	1.25 (0.20 to 3.52)	0.151*	2.40 (0.40 to 3.90)	0
Monocytes %	Median (IQR)	4.80 (3.30 to 6.40)	4.25 (3.00 to 5.20)	0.002*	4.50 (3.00 to 6.00)	4.30 (2.97 to 5.15)	0.671*	4.90 (3.02 to 6.52)	4.20 (3.10 to 5.40)	0.545*	5.20 (3.20 to 6.40)	0
Basophils %	Median (IQR)	0.30 (0.20 to 0.50)	0.30 (0.20 to 0.50)	0.080*	0.30 (0.20 to 0.50)	0.40 (0.27 to 0.52)	0.898*	0.30 (0.27 to 0.60)	0.30 (0.20 to 0.50)	0.676*	0.30 (0.20 to 0.50)	0
RBC count (million/cumm)	Median (IQR)	4.61 (4.08 to 5.14)	4.35 (3.82 to 4.87)	0.124*	4.71 (4.26 to 5.27)	4.5 (4.36 to 4.81)	0.650*	4.76 (4.38 to 5.19)	4.44 (4.10 to 4.91)	0.320*	4.83 (4.60 to 5.30)	0
Platelet count (lakh cells/cumm)	Median (IQR)	2.38 (1.87 to 3.02)	2.79 (2.21 to 3.46)	< 0.001*	2.45 (1.8 to 3.17)	3.16 (2.25 to 3.60)	0.201*	2.15 (1.80 to 2.56)	2.70 (2.23 to 3.39)	0.002*	2.09 (1.95 to 2.54)	0
Neutrophil-lymphocytes ratio (NLR)	Median (IQR)	5.80 (3.0 to 11.4)	6.0 (3.20 to 12.0)	0.749*	6.1 (3.5 to 13.2)	6.25 (2.97 to 10.97)	0.692*	5.85 (3.52 to 10.47)	5.15 (3.2 to 8.7)	0.769*	6.1 (3.0 to13.4)	0

*Kruskal-Wallis test. †Pearson Chi sq test.

LOS-Length of hospital stay, AECOPD=Acute exacerbation of chronic obstructive pulmonary diseases, NBE=No bacterial and no eosinophilia, B-Bacterial, E-Eosinophilia, and no bacterial infections, BE- bacterial infection with eosinophilia, P-pneumonia, and BC-bronchiectasis, NTS & NBMS: Non-tobacco smoke and non-biomass smoke, TS: Tobacco smoke, BMS: Biomass smoke, TS & BMS: Tobacco smoke and biomass smoke, CCI- Charlson comorbidity index, OSA-Obstructive sleep apnea, PAH-pulmonary hypertension, T1RF-Type 1 respiratory failure, T2RF-Type 2 respiratory failure, NLR-Neutrophil-lymphocytes ratio. TS & BMS-AECOPD are 100% males.

Table S4: Binomial logistic regression for the type of ward as an outcome

						95% Confidence Interval	
**Predictor**	**Estimate**	**SE**	**Z**	**p**	**Odds ratio**	**Lower**	**Upper**
Intercept	-2.4689	0.45357	-5.443	< .001	0.0847	0.0348	0.206
AECOPD-E – AECOPD-NBE	-1.5240	0.37389	-4.076	< .001	0.2178	0.1047	0.453
AECOPD-P – AECOPD-NBE	1.6841	0.16131	10.440	< .001	5.3877	3.9273	7.391
AECOPD-BC – AECOPD-NBE	1.1934	0.21762	5.484	< .001	3.2983	2.1530	5.053
AECOPD-B – AECOPD-NBE	2.3313	0.16791	13.884	< .001	10.2911	7.4052	14.302
AECOPD-BE – AECOPD-NBE	1.4305	0.32121	4.454	< .001	4.1809	2.2277	7.847
Female – Male	0.0249	0.23868	0.104	0.917	1.0252	0.6422	1.637
Age in years	-0.0100	0.00821	-1.224	0.221	0.9900	0.9742	1.006
CCI	0.2310	0.05406	4.272	< .001	1.2598	1.1331	1.401
TS-AECOPD– NTS & NBMS-AECOPD	-0.0566	0.15149	-0.374	0.709	0.9450	0.7022	1.272
BMS-AECOPD– NTS & NBMS-AECOPD	-0.1479	0.24763	-0.597	0.550	0.8625	0.5309	1.401
TS & BMS-AECOPD- NTS & NBMS-AECOPD	-0.2289	0.47840	-0.479	0.632	0.7954	0.3114	2.031

Note. Estimates represent the log odds of "Ward = 2" vs. "Ward = 1"

AECOPD=Acute exacerbation of chronic obstructive pulmonary diseases, NBE=No bacterial and no eosinophilia, B-Bacterial, E-Eosinophilia and no bacterial infections, BE-bacterial infection with eosinophilia, P-pneumonia, and BC-bronchiectasis, CCI- Charlson comorbidity index, NTS & NBMS: Non-tobacco smoke and non-biomass smoke, TS: Tobacco smoke, BMS: Biomass smoke, TS & BMS: Tobacco smoke and biomass smoke.

Table S5: Binomial logistic regression for the length of hospital stays as an outcome

						95% Confidence Interval	
**Predictor**	**Estimate**	**SE**	**Z**	**p**	**Odds ratio**	**Lower**	**Upper**
Intercept	-0.9415	0.36719	-2.564	0.010	0.390	0.190	0.801
AECOPD-E – AECOPD-NBE	-0.3402	0.15888	-2.141	0.032	0.712	0.521	0.972
AECOPD-P – AECOPD-NBE	0.7368	0.14050	5.244	< .001	2.089	1.586	2.752
AECOPD-BC – AECOPD-NBE	0.3890	0.19001	2.047	0.041	1.476	1.017	2.141
AECOPD-B – AECOPD-NBE	0.8287	0.15111	5.484	< .001	2.290	1.703	3.080
AECOPD-BE – AECOPD-NBE	0.8364	0.29375	2.847	0.004	2.308	1.298	4.105
Female – Male	0.3074	0.18995	1.618	0.106	1.360	0.937	1.973
Age in years	-0.0162	0.00676	-2.394	0.017	0.984	0.971	0.997
CCI	0.1805	0.04490	4.021	< .001	1.198	1.097	1.308
TS-AECOPD– NTS & NBMS-AECOPD	-0.0378	0.12490	-0.302	0.762	0.963	0.754	1.230
BMS-AECOPD– NTS & NBMS-AECOPD	0.0313	0.19504	0.160	0.873	1.032	0.704	1.512
TS & BMS-AECOPD-NTS & NBMS-AECOPD	-0.5076	0.47378	-1.071	0.284	0.602	0.238	1.523

Note. Estimates represent the log odds of "Length_of_hospital_stay_ = 1" vs. "Length_of_hospital_stay_ = 0"

AECOPD=Acute exacerbation of chronic obstructive pulmonary diseases, NBE=No bacterial and no eosinophilia, B-Bacterial, E-Eosinophilia and no bacterial infections, BE-bacterial infection with eosinophilia, P-pneumonia, and BC-bronchiectasis, CCI- Charlson comorbidity index, NTS & NBMS: Non-tobacco smoke and non-biomass smoke, TS: Tobacco smoke, BMS: Biomass smoke, TS & BMS: Tobacco smoke and biomass smoke.
